# Supplementary material for: Atomic scale disorder and reconstruction in bulk infinite-layer nickelates lacking superconductivity
Source: Nat Commun. 2024 Jun 14;15:5104. doi: 10.1038/s41467-024-49533-1 (PMC11178912; doi:10.1038/s41467-024-49533-1)
Supplement: Supplementary file 1 — Supplementary Information [file 41467_2024_49533_MOESM1_ESM.pdf]

Supplementary information

Atomic scale disorder and reconstruction in bulk infinite-layer nickelates lacking superconductivity

Kejun Hu[1, 4], Qing Li[2, 4], Dongsheng Song[1, 4, *], Yingze Jia[1], Zhiyao Liang[1], Shuai Wang[1], Haifeng Du[3], Hai-Hu Wen[2, *], Binghui Ge[1, *]

[1] *Information Materials and Intelligent Sensing Laboratory of Anhui Province, Anhui Key Laboratory of Magnetic Functional Materials and Devices, Institutes of Physical Science and Information Technology, Anhui University, Hefei 230601, China*

[2] *Center for Superconducting Physics and Materials, National Laboratory of Solid State Microstructures and Department of Physics, Nanjing University, Nanjing 210093, China*

[3] *Anhui Key Laboratory of Condensed Matter Physics at Extreme Conditions, High Magnetic Field Laboratory, HFIPS, Anhui, Chinese Academy of Sciences, Hefei 230031, China*

[4] *These authors contributed equally: Kejun Hu, Qing Li, Dongsheng Song*

*Corresponding author: dsong@ahu.edu.cn; bhge@ahu.edu.cn; hhwen@nju.edu.cn

This Supplementary Information contains the following sections.

Supplementary Fig. S1 -- Enlargement of SAED.

Supplementary Fig. S2 -- 4D-STEM NBED characterization.

Supplementary Fig. S3 -- Atomic resolution images of sub-regions within the interleaved area.

Supplementary Fig. S4 -- Crystalline orientation measured by TKD.

Supplementary Fig. S5 -- XPS survey spectrum of the CaH_2.

Supplementary Fig. S6 -- EDS and EELS measurements.

Supplementary Fig. S7 -- The EELS data of $Nd_{0.8}Sr_{0.2}NiO_2$ in this manuscript compared with those of Goodge, B. H.

Supplementary Fig. S8 -- Lattice parameter analysis for the stripe structure.

Supplementary Fig. S9 -- Comparison of the EELS with different orientations.

Supplementary Fig. S10 -- iDPC images simulations of the infinite layer structure.

Supplementary Fig. S11 -- The O-Ni-O bond angles in bulk $Nd_{0.8}Sr_{0.2}NiO_2$.

Supplementary Fig. S12 -- Structure characterization of bulk $Nd_{0.8}Sr_{0.2}NiO_3$ before topotactic reduction.

Supplementary Fig. S13 -- Atomic structure in bulk $Nd_{0.8}Sr_{0.2}NiO_3$ before topotactic reduction.

Supplementary Fig. S14 -- Discontinuity of Ni-O planes within the grains.

Supplementary Fig. S15 -- TEM observations of samples by grinding.

Supplementary Fig. S16 -- Effect of electron-beam under accumulated dose.

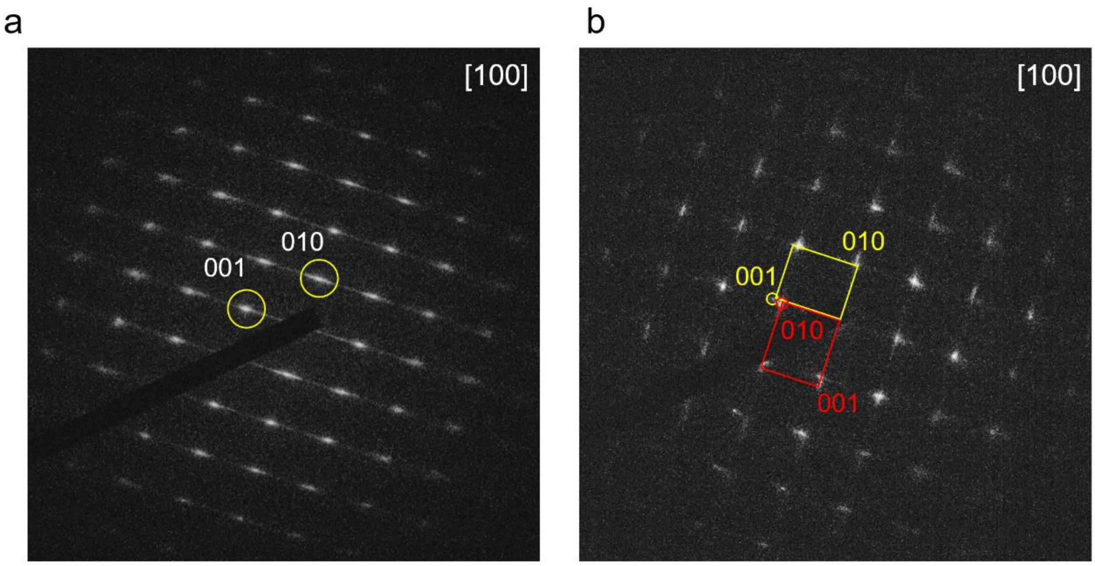

Supplementary Fig. S1 | a and **b** Enlarged views of selected area electron diffraction (SAED) in **Fig. 1e** (left) and **Fig. 1f** (right), respectively.

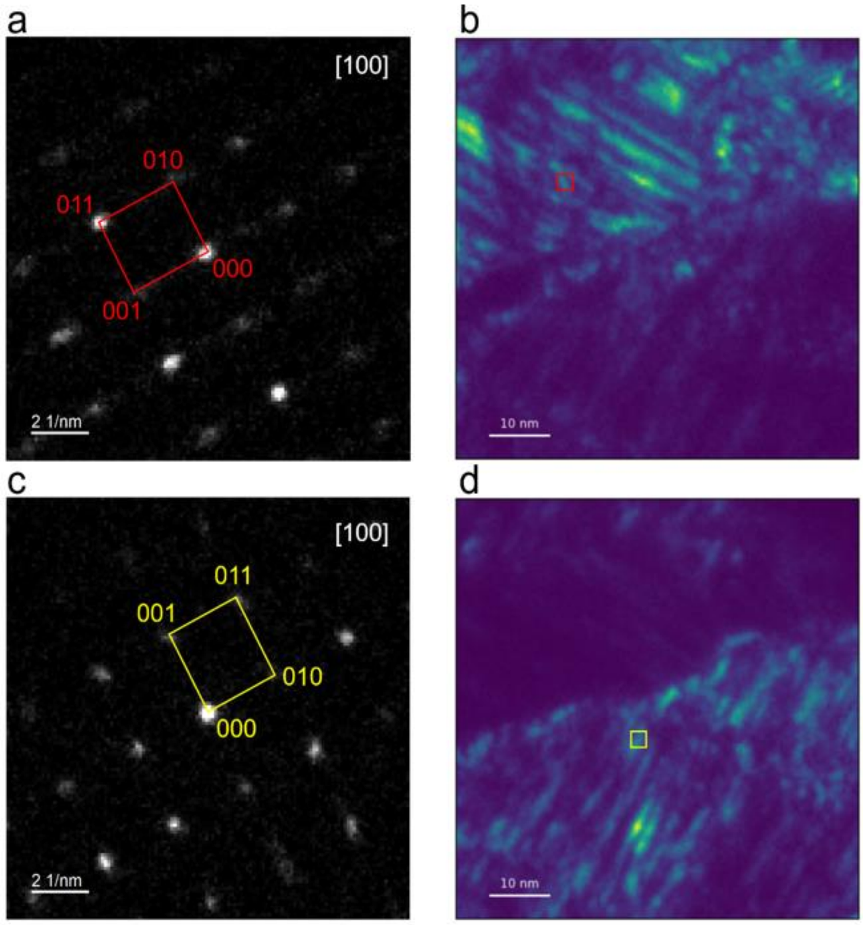

Supplementary Fig. S2 | Analysis of the interleaved regions by Scanning diffraction measurements based on the four-dimensional STEM (4D-STEM) technique. a and **c** The typical nano-beam electron diffraction patterns extracted from the two orthogonal regions in the reduced grain. **b** and **d** Virtual dark-field images reconstructed by selecting the 011 spots, respectively.

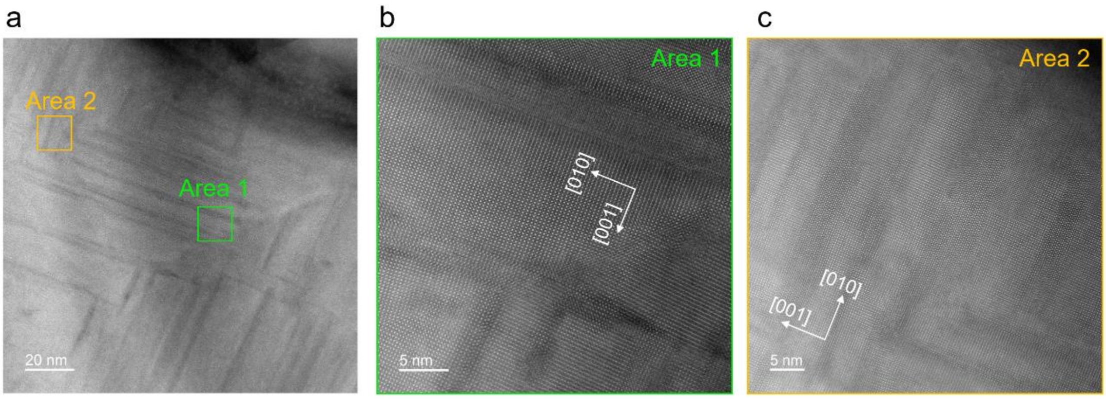

Supplementary Fig. S3 | Atomic resolution images of sub-regions within the interleaved area. **a** The HAADF image of the same grain as **Fig. 1f**. **b** and **c** The enlargement of the corresponding color box areas in **a** respectively.

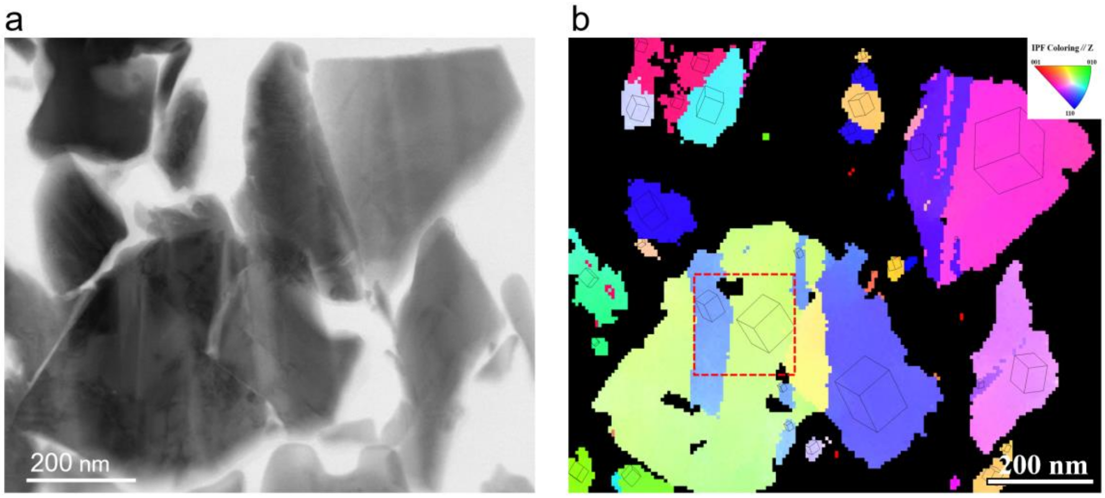

Supplementary Fig. S4 | Crystalline orientation measured by Transmission Kikuchi Diffraction (TKD). **a** The BF image of $Nd_{0.8}Sr_{0.2}NiO_2$ including multiple grains. **b** The measured crystalline orientation map corresponding to the image in **a**.

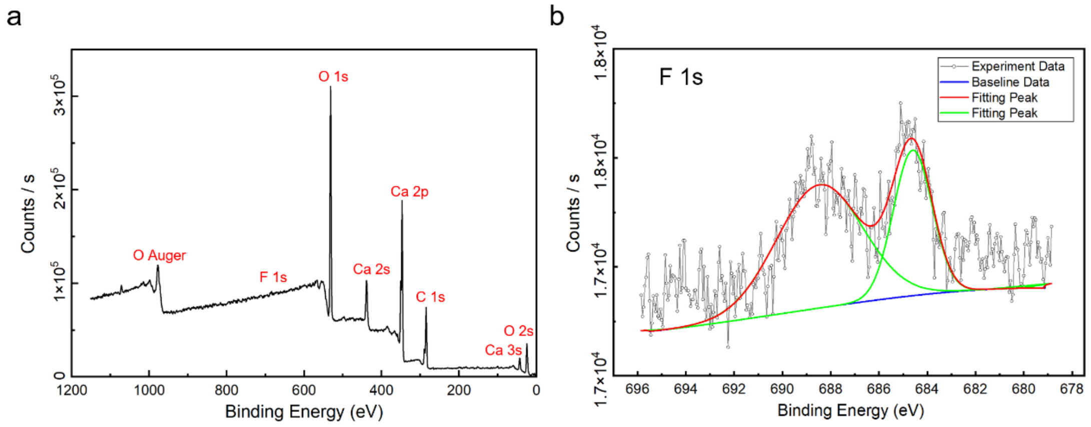

Supplementary Fig. S5 | XPS survey spectrum of the CaH_2. a XPS survey spectrum of a CaH_2 surface. **b** High-resolution XPS spectra of F(1s) obtained from the CaH_2.

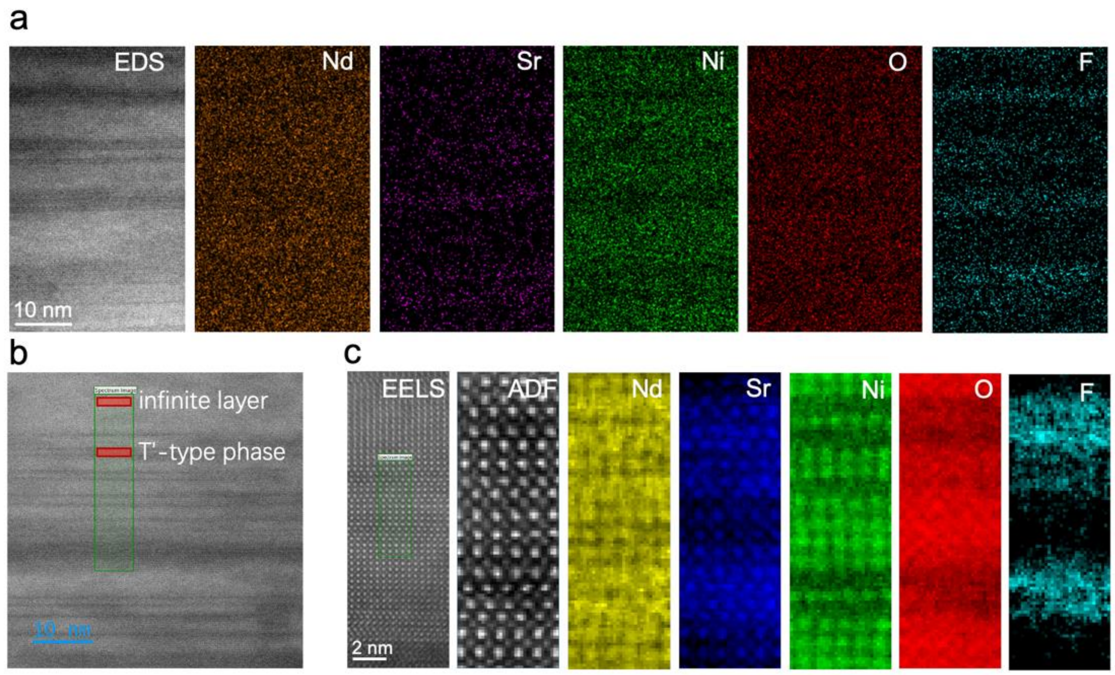

Supplementary Fig. S6 | EDS and EELS measurements. a Unfiltered EDS elemental mappings in **Fig. 3a**. **b** EELS acquisition at different locations in **Fig. 3c**. To improve the signal-to-noise ratio, integrate the signal over the regions as indicated by the red boxes. **c** EELS mapping at atomic resolution of the T′-type phases.

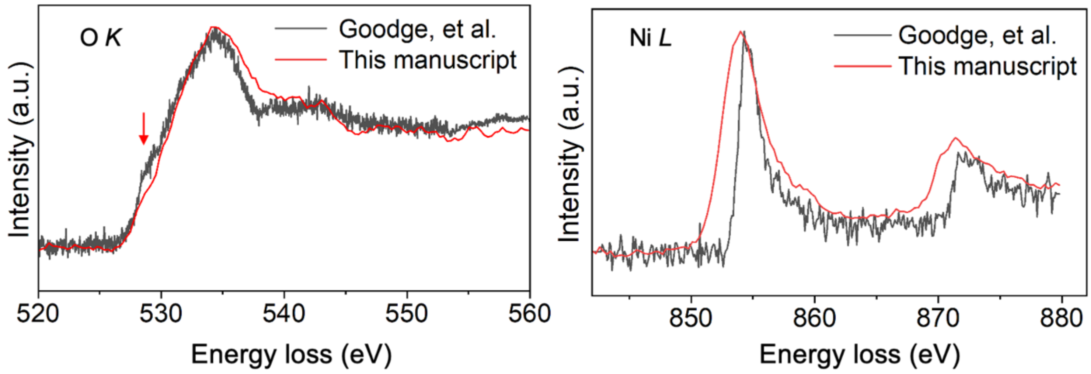

Supplementary Fig. S7 | The EELS data of $Nd_{0.8}Sr_{0.2}NiO_2$ in this manuscript compared with those of *Goodge, et al.*[31]. The weak pre-peak of O *K* edge corresponds to the underdoped level of Sr, as indicated by the red arrows. The shift of Ni $L_{3,2}$ edges towards lower energy is consistent with the lower Sr doping, despite the broadening of the peaks, which might be attributed to the relatively lower EELS energy resolution in our experiments (~ 1 eV).

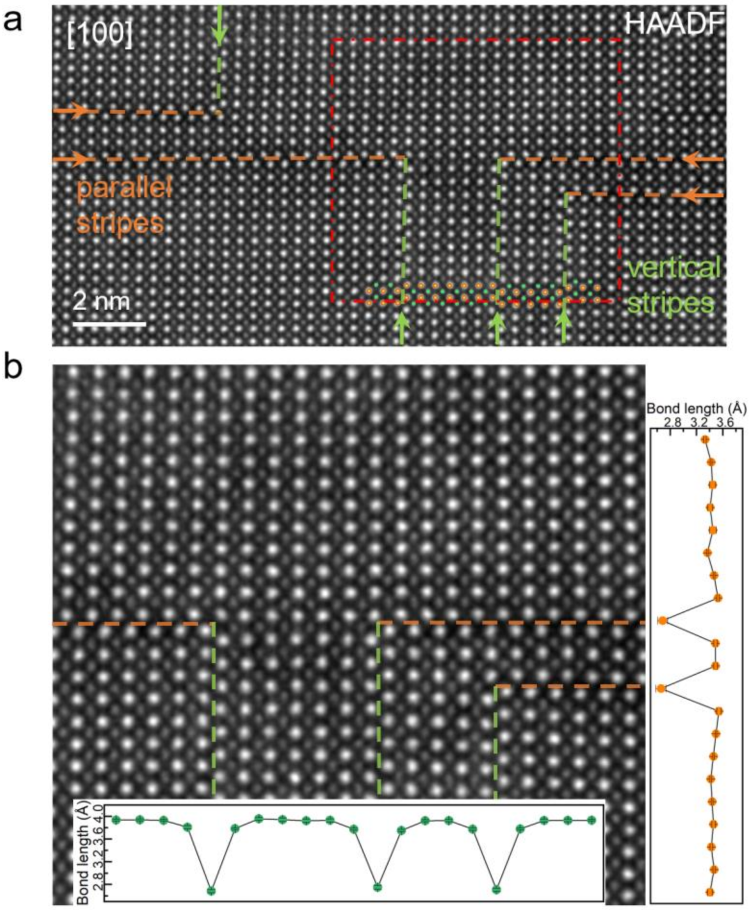

Supplementary Fig. S8 | Lattice parameter analysis for the stripe structure. a Atomic resolution HAADF-STEM images of the parallel and vertical stripes in **Fig. 4a**. **b** Averaged bond lengths of the parallel and vertical stripes measured from the red dotted box in **a**.

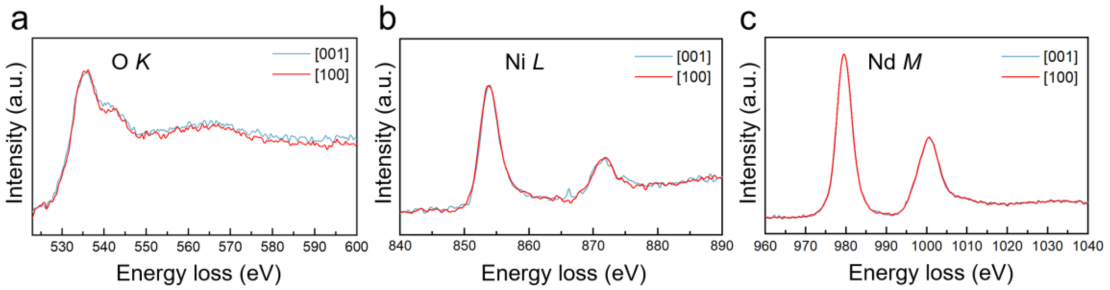

Supplementary Fig. S9 | Comparison of the EELS with different orientations. Comparison of the EELS near-edge fine structures of O-*K*, Ni-*L*, and Nd-*M* in [001] and [100] oriented grains.

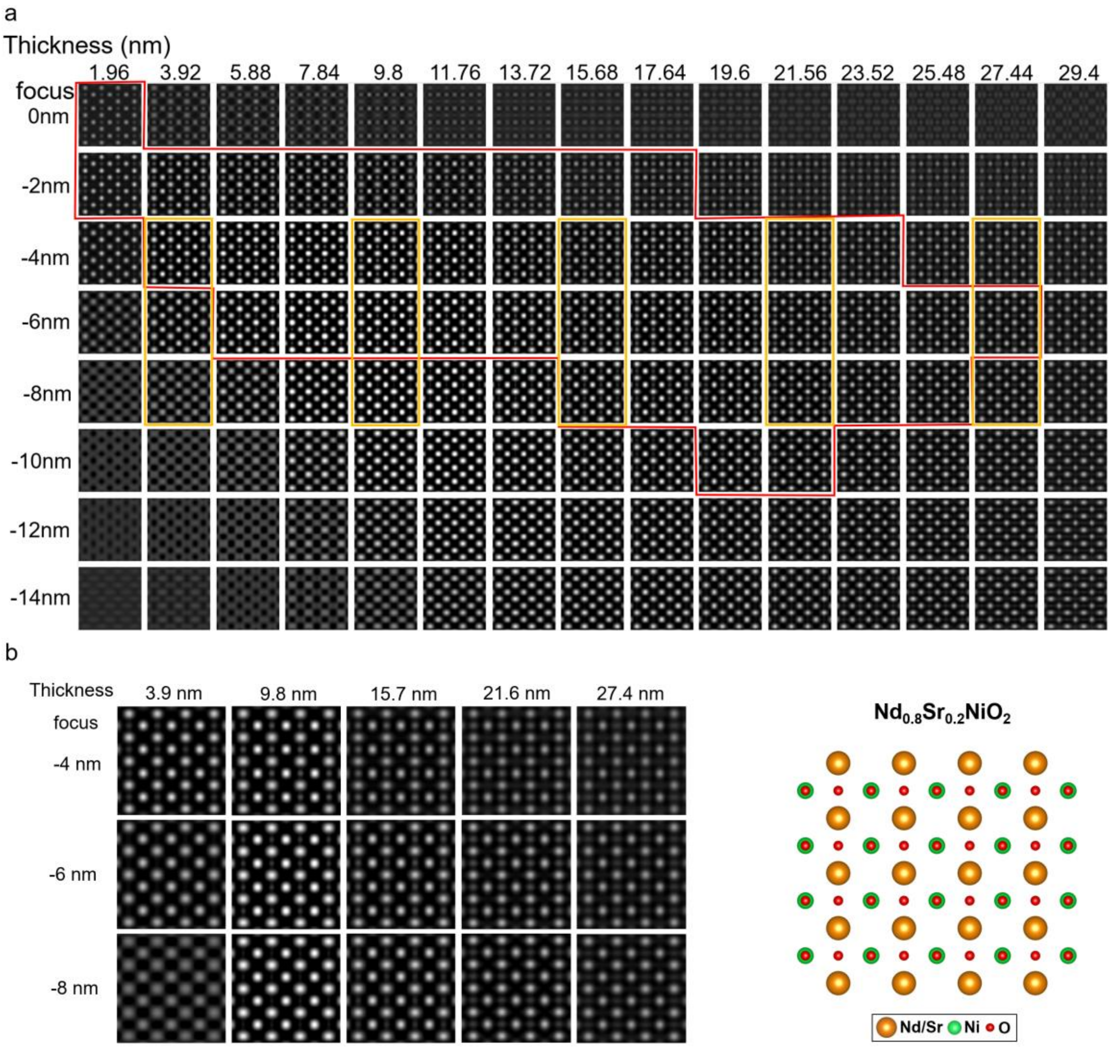

Supplementary Fig. S10 | iDPC images simulations of the infinite layer structure. a Simulated iDPC images with different thicknesses and defocus values based on the atomic model along [100] orientation. The red box shows the conditions with better imaging contrast. **b** To better show the details, some of the images are zoomed in. The atom model of the infinite layer structure is displayed on the right-hand side for comparison.

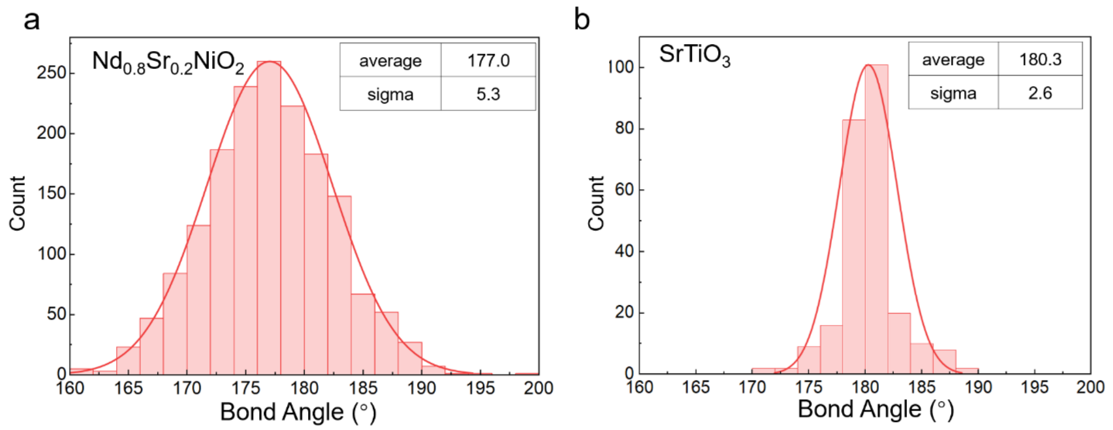

Supplementary Fig. S11 | The O-Ni-O bond angles in bulk $Nd_{0.8}Sr_{0.2}NiO_2$. a The frequency histogram of the bond angle for bulk $Nd_{0.8}Sr_{0.2}NiO_2$ measured across various regions in multiple experiments. The average bond angle deviates from 180° with a value of 177.0°±5.3°. **b** The frequency histogram of the bond angle for cubic $SrTiO_3$. For comparative purposes, we analyzed cubic $SrTiO_3$ here, where the measured bond angle is 180.3°±2.6°. Although it is difficult to maintain absolutely identical imaging conditions, e.g. aberrations, sample orientation and thickness, this comparison lends some validity to our measurements on $Nd_{0.8}Sr_{0.2}NiO_2$.

Characterization of bulk $Nd_{0.8}Sr_{0.2}NiO_3$

In STEM characterization of bulk $Nd_{0.8}Sr_{0.2}NiO_3$ before topotactic reduction, inherent defect structures similar to the RP phase were observed in the grains of its parent phase (Supplementary Fig. S12). The characteristic domain size of the precursor $Nd_{0.8}Sr_{0.2}NiO_3$ ranges from tens to hundreds of nanometers in length and several to tens of nanometers in width. These defects likely contribute to the formation of a 3D block-like structure within grains after reduction. The results of EDS mapping showed that the distribution of elements was uniform as that of the reduced samples, as shown in Supplementary Fig. S12a. This size of grains is comparable to that observed in the reduced samples. The precursor $Nd_{0.8}Sr_{0.2}NiO_3$ shows metallic properties above 10 K in Supplementary Fig. S12b. The magnifying bright-field (BF) image in Fig. S12c and Fig. S12e reveals the presence of numerous stripes within the $Nd_{0.8}Sr_{0.2}NiO_3$ grains, similar to the block-like structure in the reduced samples. The block-like configuration within the parent phase consists of alternating RP phases, each with a distinct *c*-axis orientation, as illustrated in Supplementary Fig. S13. Consequently, the defect structures present in these parent phases serve as the origin of the block-like structure observed in the $Nd_{0.8}Sr_{0.2}NiO_2$ grains after following topotactic reduction.

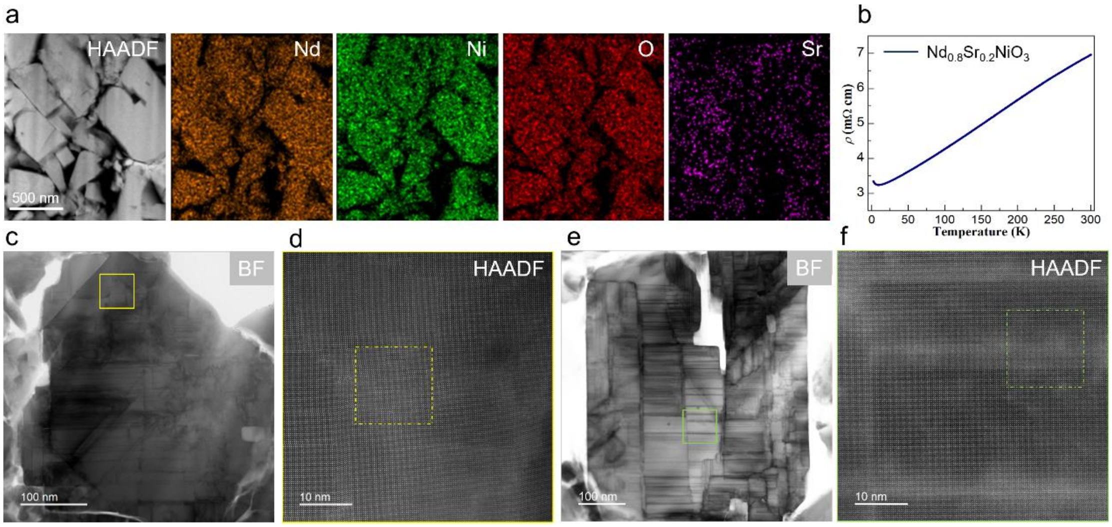

Supplementary Fig. S12 | Structure characterization of bulk $Nd_{0.8}Sr_{0.2}NiO_3$ before topotactic reduction. a EDS mapping of $Nd_{0.8}Sr_{0.2}NiO_3$ polycrystalline at low magnification. **b** Temperature dependence of resistivity for bulk $Nd_{0.8}Sr_{0.2}NiO_3$ in the range of 2-300 K. **c** and **e** High-resolution BF-STEM image of $Nd_{0.8}Sr_{0.2}NiO_3$. **d** and **f** The magnified HAADF-STEM images of marked regions in **c** and **e**.

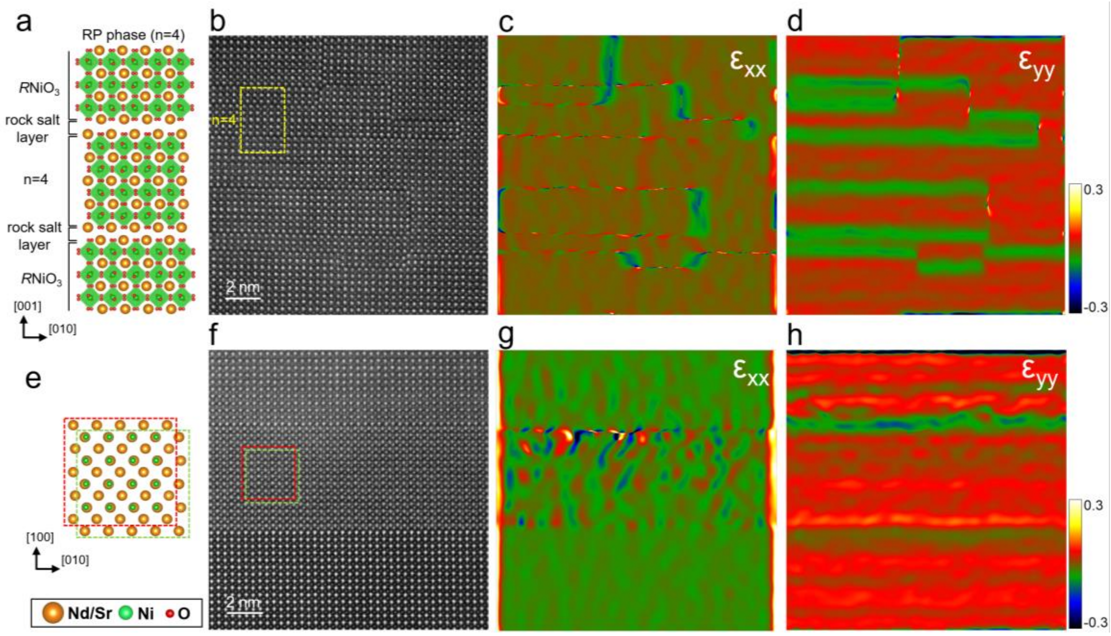

Supplementary Fig. S13 | Atomic structure in bulk $Nd_{0.8}Sr_{0.2}NiO_3$ before topotactic reduction.

a Atom model of RP phase (n = 4). **b** Atomic-resolved HAADF-STEM image of **Fig. S12d**. **c** and **d** The ε_{xx} and ε_{yy} strain maps are calculated by GPA for **b**. **e** Atom model of RP phase with *c*-axis along electron beam direction (the oxygen atoms are not shown here). **f** Atomic-resolved HAADF-STEM image of **Fig. S12f**. **g** and **h** The ε_{xx} and ε_{yy} strain maps are calculated by GPA for **f**.

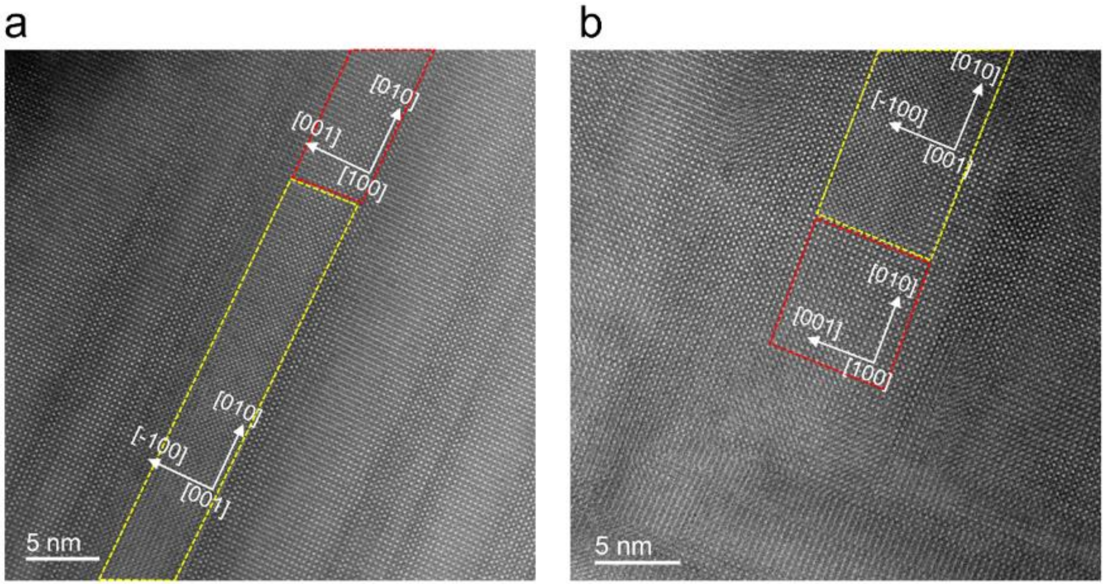

Supplementary Fig. S14 | Discontinuity of NiO_2 planes within the grains. a and **b** The red and yellow dotted boxes correspond to domains with the orientations of [100] and [001], respectively.

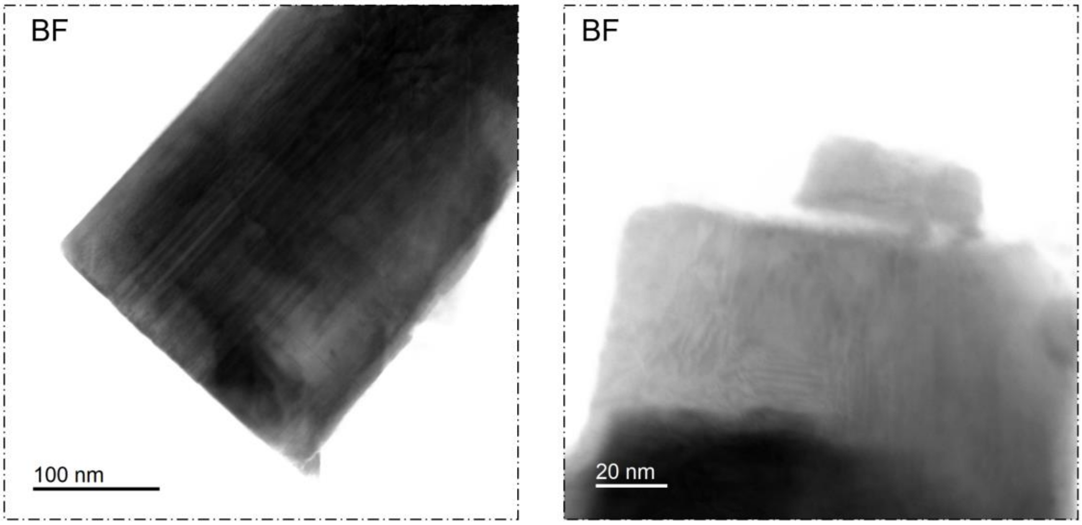

Supplementary Fig. S15 | TEM observations of samples by grinding. To rule out structural transitions or defects induced by FIB processing, we chose an alternative approach by grinding the sample, dispersing it into alcohol, and subsequently transferring it to the TEM grid. The BF images show the stripe structure and defects within $Nd_{0.8}Sr_{0.2}NiO_2$ grains.

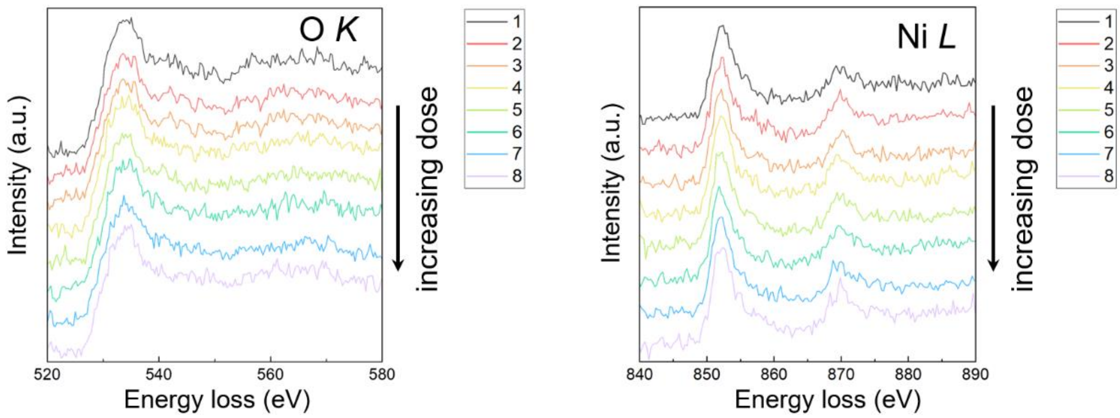

Supplementary Fig. S16 | Effect of electron-beam on the EELS O *K* and Ni *L* edges of $Nd_{0.8}Sr_{0.2}NiO_2$ under accumulated dose. The experimental parameters were consistent with those used for our EELS measurements in the main text, corresponding to an electron dose of ~ 4×10^6 $e^-/Å^2$ for each STEM-EELS mapping. The EELS mappings were conducted over the same region eight times. The averaged EELS signals are displayed. No significant modification of the O *K* and Ni *L* edges is observed.
